# Clinical and biomechanical outcome of minimal invasive and open repair of the Achilles tendon

**DOI:** 10.1186/1758-2555-3-32

**Published:** 2011-12-20

**Authors:** Alexander Pak-Hin Chan, Yue-Yan Chan, Daniel Tik-Pui Fong, Pamela Yuet-Kam Wong, Hoi-Yan Lam, Chun-Kwong Lo, Patrick Shu-Hang Yung, Kwai-Yau Fung, Kai-Ming Chan

**Affiliations:** 1 Department of Orthopaedics and Traumatology, Alice Ho Miu Ling Nethersole Hospital, Hong Kong, China; 2 Department of Orthopaedics and Traumatology, Prince of Wales Hospital, Faculty of Medicine, The Chinese University of Hong Kong, Hong Kong, China; 3 The Hong Kong Jockey Club Sports Medicine and Health Sciences Centre, Faculty of Medicine, The Chinese University of Hong Kong, Hong Kong, China; 4 Physiotherapy Department, Alice Ho Miu Ling Nethersole Hospital, Hong Kong, China

## Abstract

**Introduction:**

With evolutions in surgical techniques, minimally invasive surgical (MIS) repair with Achillon applicator has been introduced. However, there is still a lack of literature to investigate into the clinical merits of MIS over open surgery. This study aims to investigate the correlation between clinical outcome, gait analysis and biomechanical properties comparing both surgical methods.

**Materials and methods:**

A single centre retrospective review on all the consecutive operated patients between January 2004 and December 2008 was performed. Twenty-six patients (19 male and 7 female; age 40.4 ± 9.2 years) had experienced a complete Achilles tendon rupture with operative repair. Nineteen of the patients, 10 MIS versus 9 open repairs (13 men with a mean age of 40.54 ± 10.43 (range 23-62 yrs) and 6 women with a mean age of 45.33 ± 7.71 (range 35-57 yrs) were further invited to attend a thorough clinical assessment using Holz's scale and biomechanical evaluation at a mean of 25.3 months after operation. This study utilized the Cybex II isokinetic dynamometer to assess the isokinetic peak force of plantar-flexion and dorsiflexion of both ankles. The patients were also invited to return to our Gait Laboratory for analysis. The eight-infrared camera motion capture system (VICON, UK) was utilized for the acquisition of kinematic variables. Their anthropometric data was measured according to the Davis and coworkers' standard.

**Results:**

The mean operative time and length of hospital stay were shorter in the MIS group. The operative time was 54.55 ± 15.15 minutes versus 68.80 ± 18.23 minutes of the MIS group and Open group respectively (p = 0.045), whereas length of stay was 3.36 ± 1.21 days versus 6.40 ± 3.70 days respectively (p = 0.039). There is statistically significant decrease (p = 0.005) in incision length in MIS group than the open surgery group, 3.23 ± 1.10 cm versus 9.64 ± 2.55 cm respectively. Both groups attained similar Holz's scores, 11.70 ± 0.95 versus 12.0 ± 1.50 respectively (p = 0.262). The mean percentage stance time of the injured leg for MIS patient was 58.44% while the mean percentage stance time of the injured leg for patients with open repair was 56.57%. T-test has shown there were no significance differences between the results of the two groups of patients. The loss of peak torque and total work done with respect to the injured side were similar between the MIS and open group.

**Discussion and conclusion:**

MIS using Achillon method can achieve smaller incisions, shorter operative time and hospital stay. There is no statistical significance difference in clinical outcome, the stance time to strike time ratio and biomechanical properties on the leg receiving Achilles tendon repair using MIS method and open surgery.

## Background

Achilles tendon rupture most commonly occurs during recreational sports that require bursts of jumping, pivoting, and running. The incidence rate of Achilles tendon ruptures accounted for an overall mean of 8.3 ruptures per 100,000 people [1]. The mean age for an Achilles tendon rupture was 40.6 years for men and 44.5 years for women [1]. The most common sports activities causing Achilles tendon rupture were soccer (28%), tennis (12%), volleyball (7%), squash (7%), athletics (7%) and skiing (3%). 25% of the tendon ruptures were non-sports related [2]. Achilles tendon ruptures accounted for 34.6% of all badminton injuries amongst recreational players and beginners [3].

The recovery course can be variable and prevent patient to return to sports to the full. Operation is the treatment of the choice to enhance tendon approximation and tendon healing but conventional open repair can bring complications including wound infections, skin tethering, sural nerve damage, hypertrophic scars are not uncommon, accounting for 4-19% of patients [2,4-6]. Some centres adopted conservative management with casting instead of operations to avoid these surgical complications. However, from a recent study published by Keating JF [7], surgeries seemed to play a role in the early post-operative phase. The documented re-rupture rate occurred in two of 37 patients was 5% (2 out of 37) in the operative group and 10% (4 out of 39) in the non-operative group by casting, which was not statistically significant (p = 0.68). At three months, the operative group enjoyed a slightly greater range of plantar flexion and dorsiflexion of the ankle (statistically not significant), less peak torque difference of plantar flexion compared with the normal side than the non-operative group (47% vs 61%, respectively, p < 0.005) as well as significantly better mean Short Musculoskeletal Function Assessment (SMFA) scores than the non-operative group (15 vs 20, respectively, p < 0.03). However, the study was unable to show a functional benefit from surgery for patients compared with conservative treatment in plaster after 3 months and up to 1 year.

Minimally invasive surgical (MIS) repair with Achillon applicator has been proven to be a safe and effective method for the management of for Achilles tendon rupture. The merits of minimally invasive surgical repair with Achillon applicator include limiting exposure of tendon, decreasing chance of sural nerve injury, reducing amount of suture material, allowing closure of paratenon and enhancing blood supply to facilitate maximal wound healing [8,9]. These can explain the lower propensity of wound infections after minimally invasive method then conventional open method [8,9]. Since the year 2007, MIS method with Achillon applicator has been introduced in the Department of Orthopaedics and Traumatology, Alice Ho Miu Ling Nethersole Hospital (AHNH).

However, parameters assessing the functional recovery after an Achilles tendon rupture are few and not well investigated. It has been shown that an incomplete functional recovery exists despite good results in terms of overall outcome and patients' satisfaction [10-12]. Of note, these reported literatures thus far are primarily based on the Western population. There is still a lack of literature to investigate on the merits of Achillon method over conventional open surgery in terms of functional outcome.

### Objective and Hypothesis

The purpose of this study is to retrospectively investigate and compare MIS repair with Achillon device with open method in terms of plantar flexor muscle-tendon unit properties and the gait pattern in Chinese patients who underwent surgical repair of Achilles tendon rupture, by means of a multidisciplinary approach with clinical assessment, biomechanical evaluation and gait analysis.

The hypothesis of this study is that patients from the MIS group can have shorter skin incisions, less wound infection, shorter operative time and shorter hospital stay than Open group.

## Research design and methodology

A single centre retrospective case series study on all the consecutive patients undergoing surgical repair of primary Achilles tendon rupture between January 2004 and December 2008 in AHNH was performed. The patients were divided into two groups, the MIS and the other being the open repair group, for further comparison. Patients' age, gender, laterality, type of sport involved, mechanism of injury, occupation, symptoms at presentation, dominant injury versus non-dominant limb injury, physical findings, radiological findings, operative findings, procedure undertaken, operative duration, surgical complications (wound infection, rerupture), clinical outcome and length of hospital stay were retrieved from medical records. Patients were further invited through telephone to return to hospital for further single-observer clinical assessment, single-observer gait analysis and single-observer biomechanical study.

Exclusion criteria of this study included patients not being able to attend the assessments and those who did not undergo operative treatment for Achilles tendon rupture. From the data retrieved from our department, all the patients admitted during the study period were surgically fit for operation. After discussing treatment options of operative versus non-operative management, all the patients opted for surgical repair for the tendon ruptures.

### Operation

The operations were performed under general anaesthesia in a prone position. A tourniquet was always used over the thigh with exsanguination. A single dose of 1 g cephalosporin prophylactic antibiotic was administered on induction.

#### Open Technique

A straight skin incision starting from the medial aspect of the heel up to the middle of the calf, preserving the lesser saphenous vein and the sural nerve, was made [2]. The paratenon was then carefully dissected. The tendon was adapted with Ethibond sutures (non-absorbable) using the Krackow method in 20° plantar flexion of the ankle.

#### Minimally Invasive Repair

The operations were performed using the Achillon suture guide as first described by Assal et al, with the use of a U-shaped device with 4 limbs for tendon approximation.^8 ^A stab incision through the paratenon was made over the ruptured site, with both ends exposed. The haematoma was debrided. 3 Ethibond 2 sutures were passed through the holes of Achillon applicator, and tightened at full plantarflexion. The paratenon was then closed with Vicryl sutures with skin closed in layers.

### Postoperative care and Rehabilitation

The postoperative management of all the subjects were the same. For the first 2 weeks after surgery, a short-leg cast was applied with the ankle in 20° equines position. The cast was then changed to an ankle walker for 2 weeks, and patients were allowed to increase weight-bearing gradually. Sport activities were not allowed for 3 months after the operation. Patients were followed up at 2 weeks, then monthly.

### Clinical Assessment

This was performed by the principal investigator of this study, an Orthopaedic surgeon. Patients were requested to sign an informed consent form for participation of this study. A questionnaire had to be further filled in, particularly document dominant versus non-dominant limb injury. The dominant limb was defined as the limb preferred to execute a manipulative or mobilizing action while the non-dominant limb provide stabilizing support [13]. The recruited patients were assessed on surgical incision lengths and on Holz's scale [14].

### Gait analysis

Gait analysis was performed by the same investigator, a Research Assistant from the Department of Orthopaedics and Traumatology, the Chinese University of Hong Kong, using the eight-infrared camera motion capture system (VICON, UK) at 120 Hz for the acquisition of kinematic variables. Two Kistler platforms (Kistler Instruments, Winterthur, Switzerland) were used to acquire ground reaction forces. Subjects were instructed to walk at a self-selected speed along a level surface approximately 10 m in length and practise until they could consistently and naturally make contact with both the force platforms. Five trials were acquired for each subject. The stride duration, stance phase and swing phase duration, as well as step length of both the injured and un-injured side were measured.

### Biomechanical evaluation

The Cybex II+ isokinetic dynamometer with dual channel recorder and Cybex data reduction computer (CDRC), Upper Body Exercise Table (UBXT), and plantarflexion footplate were used in this study (Cybex, Division of Lumex, Ronkonkoma, New York, USA) to assess the final isokinetic peak force of plantar flexion of both ankles was applied when the patients had returned to their pre-injury activity level [15].

All subjects were required to begin testing with a prescribed warm-up programme. The Cybex set-up and positioning for plantar-flexion were in accordance with the Cybex isolated joint testing manual and with the knee in a fully extended position (0°). All adjustments were made by the same Physiotherapist of Physiotherapy Department, AHNH. Three submaximal and two maximal peak force records were taken from the injured and uninjured ankle by the same physiotherapist to eliminate inter-observer errors.

Testings were carried out at speeds of 60° s^-1 ^and 180° s^-1^. Testing at 60° s^-1 ^included five repetitions of exercise, while 25 consecutive repetitions were performed at 180° s^-1^. The isokinetic measures tested and utilized for the statistical analysis included peak torque at 60° s^-1 ^and 180° s^-1 ^and total work (TW) of the injured as well as un-injured side. In order to measure the difference of the ankle flexors, torque ratio and work ratio (dorsiflexor/plantar-flexor) were calculated. Ankle dorsiflexion and plantarflexion range were also measured. Peak torque and TW are given in Newton-metres (Nm) and AP in watts.

### Statistical Analysis

With the assumption that the parameters sampled from populations from normal distribution, the variances of the MIS and Open groups are unknown, the parameters are simple, randomly selected and independent, the 2-independent sample t-test was applied. Let a = 0.05, and 95% Confidence Interval for the mean difference. All the data collected upon assessments were further subjected to the checking of normality of data.

### Clinical Ethics

This research protocol was submitted to the Joint Chinese University of Hong Kong-New Territories East Cluster Clinical Research Ethics Committee (CREC). The ethics approval of the research protocol was adopted from the CREC with reference number CRE-2009. 197 in May 2009, titled "Gait analysis following surgery for Achilles tendon rupture: Comparison of open repair with minimally invasive repair". Biomechanical studies and gait analysis were then conducted in compliance with the research regulations.

## Results

A total of 26 patients (19 male and 7 female) were identified within the study period and mean age of 42.1 years (range 23-62 years). 15 patients belonged to the Open group while another 11 patients were operated by MIS technique. Three of the patients underwent ultrasonographic investigation demonstrating a gap sign of the Achilles tendon, corresponding to complete rupture of the tendon. All the rest 19 patients were operated based on clinical findings of gap sign and also positive Simmond's test.

A large majority of patients (42.3%) were recreational badminton players, followed by soccer (19.2%), basketball (11.5%), volleyball (3.9%) and jogging (3.9%) (Table 1). Forceful plantarflexion (20/22) was the most common mechanism of injury.

**Table 1:** Sports Involved and Mechanisms of Injury

	Forceful Plantarflexion	Direct Contusion	Cumulative Frequency
**Badminton**	11	-	11
**Soccer**	4	1	5
**Basketball**	2	1	3
**Volleyball**	1	-	1
**Jogging**	1	-	1
**Trauma**	1	4	5

**Total**	20	6	26

Through telephone invitation, 19 of the 26 patients (13 male and 6 female), mean age of 42.05 years (range 23-62 years) enrolled for further clinical assessment, gait analysis and biomechanical assessment. This included 10 MIS group patients and 9 Open group patients. Seven patients were excluded because 6 patients were lost due to change of contact telephone number and address, and 1 patient was unable to attend due to work in China. The mean age of the 10 MIS group patients was 41.70 years (range 29-57 years) whereas the mean age of the 9 Open group patients was 42.44 years (range 23-62 years). On questionnaire survey, 6 of the recruited 19 patients had seen bone setter with a mean 4.67 days delay (range 0-10 days) between injury and admission to our hospital. The remaining 10 patients, not having been seen by bone setter, had a mean 2.77 days delay (range 0-30, mode 0) between the injury and admission to our hospital.

The mean days between injuries and admission to our hospital were 5.20 days (range 0-30 days) versus 1.33 days (range 0-4 days) in the MIS group and Open group respectively. The documented number of days between injuries and operations were 8.90 days (range 3-30 days) and 5.78 (range 1-8 days) respectively. The interval between operation and clinical assessment was 12.00 months (Range 6-19 months) and 40 months (Range 6-62 months) for the two groups respectively.

Not all patients presented with pop sound during injury. Combination weakness and pop sound were the most common initial symptoms, accounting for 31.6% (6/19) patients (Table 2). Less common complaints were pain and weakness (21.0%, 4/19), pain and pop sound (15.8%, 3/19), pain together with weakness and pop sound (15.8%, 3/19), weakness (10.5%, 2/19) and pain (5.3%, 2/19). For complaints at hospital, 31.6% patients presented with pain and weakness, 21.0% with weakness and pop sound, 15.8% presented with weakness or pain and pop sound, 10.5% with combination of pain, weakness and pop sound, 5.3% with pain respectively.

**Table 2:** Symptomatology at injury and when attending hospital.

	Initial complaints		Complaints at hospital	
	**MIS (10)**	**Open (9)**	**MIS (10)**	**Open (9)**

**Pain**	1	-	1	-
**Weakness**	1	1	1	2
**Pop sound**	-	-	-	-
**Pain+ Weakness**	2	2	4	2
**Pain+ Pop sound**	2	1	2	1
**Weakness + Pop sound**	4	2	2	2
**Pain+Weakness+Pop sound**	-	3	-	2

Sixty percent (6/10) of the MIS group suffered from dominant side injury while only 33.3% (3/9) of patients in the OPEN group suffered from ruptured Achilles tendon on the dominant side, with p value being equal 0.370 on Fisher's Exact Test. As the study population was less than 50, Shapiro-Wilk test for normality was utilized for normality testing. With a = 0.05, Effect size d = 0.5, with MIS group number being equal to 10, Open group equal to 9, the power of the study was 0.1769673 by using G*Power 3.1.2 software (Heinrich Heine Universität Düsseldorf).

### Primary outcomes

Patients from the MIS group benefited from shorter operative time, 54.55 ± 15.15 minutes (range 35-90 minutes), with the contrary of 68.80 ± 18.23 minutes (range 40-100 minutes) in the Open group, with statistically significance (p = 0.045) (Table 3). The average number of days of hospital stay were noted to be shorter amongst the MIS group than the Open group, 3.36 ± 1.21 days (range 2-5 days) versus 6.40 ± 3.70 days (range 3-16 days) respectively (p = 0.039) (Table 3).

**Table 3:** Total Operative Time, Length of Hospital stay and Primary Outcomes comparing MIS and Open group.

	MIS (11)	Open (15)	Significance test
Total Operative Time (Min)	54. 6 ± 15.2 (Range 35-90)	68.8 ± 18.2 (Range 40-100)	p = 0.045 CI -28.196 to -0.314

Length of Hospital Stay (Days)	3.4 ± 1.2 (Range 2-5)	6.4 ± 3.7 (Range 3-16)	p = 0.039 CI -5.437 to -0.645

	**MIS (10)**	**Open (9)**	**Significance Test**

**Incision length**	3.2 ± 1.0 (1.8-5.0)	9.6 ± 2.6 (5.8-12.0)	p = 0.005 CI -8.259 to -4.570

**Holtz's scale**	11.7 ± 0.95 (11-14)	12.0 ± 1.50 (10-14)	p = 0.262 CI -1.501 to 0.901

**Follow-up duration (months)**	6.1 ± 2.6 (Range 2 - 12)	6.6 ± 2.5 (Range 4 - 12)	p = 0.867 CI -2.912 to 2.000

**Physiotherapy duration (months)**	3.6 ± 1.0 (Range 2 - 5)	4.6 ± 1.9 (Range 3 - 9)	p = 0.169 CI -2.417 to 0.506

As for the postoperative complications, 1 female patient of the Open group with history of hypertension but no diabetes mellitus suffered from superficial wound infection which was treated with intravenous antibiotics. However, there was no documented deep wound infection for both the MIS and Open groups. Superficial wound infection accounted for an overall complication rate of 3.85%. Another male patient from the Open group suffered from a traumatic rerupture of Achilles tendon in a road traffic accident 6 months after the initial injury, requiring reconstruction operation. Otherwise, there was no spontaneous rerupture.

On clinical measurements, the MIS group benefited from shorter surgical wound incisions, with a mean length of 3.23 ± 1.03 cm (range 1.8-5.0 cm) versus a mean length of 9.64 ± 2.55 cm (range 5.8-12.0 cm) in the Open group, with statistical significance difference (p = 0.005) (Table 3). However, the mean Holtz's scale (a common parameter in assessing functional outcome after surgical repair in Achilles tendon rupture) in the MIS group and the Open group were similar, with a mean value of 11.70 ± 0.95 and 12.00 ± 1.5 respectively, p = 0.262. In addition, it was also noted that the duration of follow-up and physiotherapy was shorter in the MIS group than the Open group, with mean follow-up 6.10 ± 2.60 months (range 2-12 months) versus 6.56 ± 2.50 months (range 4-12 months) (p = 0.867); mean physiotherapy duration of 3.60 ± 0.97 months (range 2-5 months) versus 4.56 ± 1.94 months (range 3-9 months) (p = 0.169) (Table 3). Only one patient in the Open group dropped out from follow-up (11.1% of the Open group) while all the other patients attended the clinical sessions as advised.

### Gait Analysis

Gait analysis showed comparable stance duration of the injured leg and the uninjured side for both MIS and Open groups: MIS group- 1.121 second versus 1.116 second respectively; Open group- 1.051 second versus 1.057 second respectively.

The mean percentage stance time of the injured leg for MIS patient was 58.44% while the mean percentage stance time of the injured leg for patients with open repair was 56.57% (Table 4). Both the stance time possession of the injured leg and the uninjured leg within the MIS group and Open group respectively was similar: 58.44% versus 58.79% in the MIS group; 56.57% versus 56.73% in the Open group. T-test has shown there were no significance differences (p = 0.065) between the results of the two groups of patients.

**Table 4:** Stance Phase and Swing Phase proportion in both injured leg and uninjured leg, comparing MIS and Open group.

		MIS (10)%	Open (9)%	Significance test
**Stance Phase**	**Injured side**	58.4	56.6	p = 0.065
	**Un-injured side**	58.8	56.7	

**Swing Phase**	**Injured side**	41.6	43.4	
	**Un-injured side**	41.2	43.3	

The mean step length of the injured leg and the uninjured leg was similar in the MIS group, 1.122 ± 0.102 m (range 1.029-1.202 m) versus 1.113 ± 0.124 m (range 1.044-1.172 m) respectively (p = 0.378) (Table 5). The mean step length of the Open group for the injured side and the normal side was 1.214 ± 0.177 m (range 1.038-1.572 m) versus 1.220 ± 0.169 m (range 1.033-1.538 m) respectively (p = 0.378).

**Table 5:** Step length (m) in both injured leg and uninjured leg, comparing MIS and Open group.

	MIS (10)	Open (9)	Significance test
**Injured side**	1.1 SD 0.1 (1.029-1.202)	1.2 SD 0.2 (1.038-1.572)	p = 0.378
**Un-injured side**	1.1 SD 0.1 (1.044-1.172)	1.2 SD 0.2 (1.033-1.538)	

### Biomechanical Analysis

The maximal range of ankle movement measured showed similar findings with respect to plantarflexion as well as dorsiflexion were similar between the MIS and Open groups (Table 6). Mean maximal plantarflexion was 34.90° for the MIS group and 36.56° for the Open group (p = 0.803). Whereas mean maximal dorsiflexion was 18.50° and 16.89° for the MIS and Open groups respectively (p = 0.620).

**Table 6:** Maximal ankle range of movement of the injured leg, comparing MIS and Open group.

	MIS (10)	Open (9)	Significance Test
**Mean Plantarflexion (°)**	34.9 ± 5.3 (Range 23 - 42)	36.6 ± 5.8 (Range 24 - 45)	p = 0.803 CI -7.002 to 3.691
**Mean Dorsiflexion (°)**	18.5 ± 3.8 (Range 11 - 24)	16.9 ± 2.9 (Range 13 - 22)	p = 0.620 CI -1.709 to 4.931

**Figure 1:**
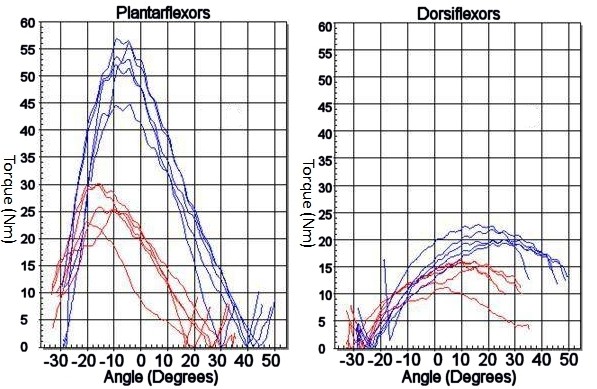
**A patient with injured right ankle (Blue line) demonstrating only half of the peak torque of the uninjured left side (Red line) on plantarflexion but similar output on dorsiflexion in the Open Group**.

**Figure 2:**
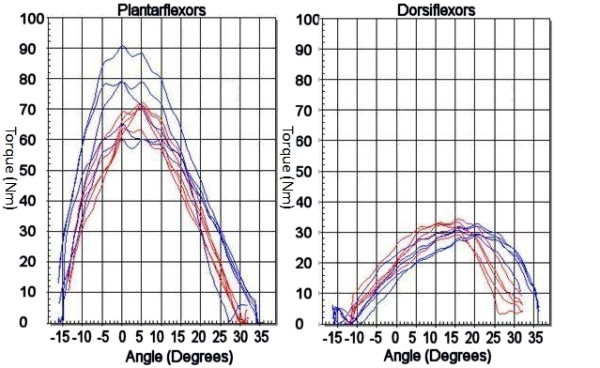
**Another patient in the MIS group demonstrating similar peak torque of the injured left ankle (Red line) as compared to the uninjured right side in both plantarflexion and dorsiflexion**.

The loss of peak torque and total work done with respect to the injured side were similar between the MIS and open groups (Table 7 and 8). For the mean peak torque in plantarflexion at 60° s^-1^, the value of the injured side was 74.78% of the uninjured side in the MIS group as compared to 82.04% of the uninjured side in the Open group (p = 0.664). At 180° s^-1^, the peak torque of the injured side was 88.1% of the unaffected side in plantarflexion in the MIS group and 96.0% in the Open group (p = 0.896). For the mean peak torque in dorsiflexion at 60° s^-1^, the injured side showed 105.11% of the uninjured side for the MIS group and 86.46% for the Open group (p = 0.348). At 180° s^-1^, the peak torque in dorsiflexion of the injured leg was 92.5% of the normal side in the MIS group and 84.2% in the Open group (p = 0.493). An example of the peak torque output curves are put up in Figure 1-2. For mean total work done in plantarflexion, the injured leg showed a value of 73.56% of the uninjured side amongst the MIS group, 72.38% in the Open group (p = 0.275) (Table 8). In dorsiflexion, the mean total work done of the injured side was 89.55% of the uninjured in the MIS group, while 76.16% in the Open group (p = 0.474).

**Table 7:** Peak Torque (Newton-Meters) in both injured leg and uninjured leg, comparing MIS and Open group.

		MIS (10) 60° s^-1^	Open (9) 60° s^-1^	MIS (10) 180° s^-1^	Open (9) 180° s^-1^
**Plantarflexion**	**Injured side (Injured side/Un-injured side%)**	42.4 (74.8%)	45.7 (82.0%)	22.3 (88.1%)	24.1 (96.0%)

**Significance test**		p = 0.664 CI -24.172 to 17.639		p = 0.896 CI -12.375 to 8.753	

	**Un-injured side**	56.7	55.7	25.3	25.1

**Significance test**		p = 0.308 CI -21.269 to 23.336		p = 0.493 CI -8.993 to 9.370	

**Dorsiflexion**	**Injured side (Injured side/Un-injured side%)**	24.7 (105.1%)	20.6 (86.5%)	13.6 (92.5%)	13.0 (84.2%)

**Significance test**		p = 0.348 CI -5.261 to 13.550		p = 0.424 CI -3.900 to 5.100	

	**Un-injured side**	23.5	23.8	14.7	15.4

**Significance test**		p = 0.040 CI -7.425 to 6.869		p = 0.263 CI -4.846 to 3.357	

**Table 8:** Total Work in both injured leg and uninjured leg, comparing MIS and Open group.

		MIS (10)	Open (9)	Significance test
**Plantarflexion**	**Injured side (Injured side/Un-injured side%)**	156.6 (73.56%)	219.2 (72.38%)	p = 0.275 CI -136.523 to 11.279
	**Un-injured side**	212.9	302.9	p = 0.706 CI -192.480 to 12.502

**Dorsiflexion**	**Injured side (Injured side/Un-injured side%)**	132.8 (89.55%)	131.0 (76.16%)	p = 0.474 CI -60.514 to 64.114
	**Un-injured side**	148.3	172.0	p = 0.295 CI -98.336 to 50.936

## Discussion

MIS technique using Achillon applicator has fulfilled the goal of MIS with shorter wound incisions, shorter operative time and length of hospital stay in our study. However, the study population is small. A better study would be a prospective cohort study. In a regional hospital, the number of cases of ruptured Achilles tendon with operation done was less than 10 per year, the accumulation and collection of adequate cases for analysis might took years. Besides, there was no standardized interval between operation and postoperative assessments. As listed previously, the interval between operation and assessments of the Open group was much shorter than the MIS group. The Open group has stopped the rehabilitative physiotherapy long before the conduction of the assessments. This might bring systematic bias to the study results. With regard to the reasons above, a multi-centre prospective cohort study has been carried out to better delineate the benefits of MIS surgery over Open surgery.

## Conclusions

The mean operative time and length of hospital stay were shorter in the MIS group. The operative time was 54.55 ± 15.15 minutes versus 68.80 ± 18.23 minutes of the MIS group and Open group respectively (p = 0.045), whereas length of stay was 3.36 ± 1.21 days versus 6.40 ± 3.70 days respectively (p = 0.039). There is statistically significant decrease (p = 0.005) in incision length in MIS group than the open surgery group, 3.23 ± 1.10 cm versus 9.64 ± 2.55 cm respectively. Both groups attained similar Holz's scores, 11.70 ± 0.95 versus 12.0 ± 1.50 respectively (p = 0.262). The mean percentage stance time of the injured leg for MIS patient was 58.44% while the mean percentage stance time of the injured leg for patients with open repair was 56.57%. T-test has shown there were no significance differences between the results of the two groups of patients. The loss of peak torque and total work done with respect to the injured side were similar between the MIS and open group.

## Competing interests

The authors declare that they have no competing interests.

## Authors' contributions

CAPH, CYY, FDTP, LHY, LCK, YPSH, FKY, CHAN KM were involved in the study design and coordination. LHY, LCK and CAPH were involved in the operative treatments of the patients. CAPH conducted the medical record retrievals, establishment of questionnaire and clinical assessments. CYY carried out gait analysis and WONG PY carried out the biomechanical assessments. CAPH, CYY, FDTP conducted the statistical analysis. All authors read and approved the final manuscript.
